# Different physiological stages and breeding systems related to the variability of meat quality of indigenous Pantaneiro sheep

**DOI:** 10.1371/journal.pone.0191668

**Published:** 2018-02-12

**Authors:** Jéssica de Oliveira Monteschio, Poliana Campos Burin, Ariadne Patricia Leonardo, Daiane Aparecido Fausto, Adrielly Lais Alves da Silva, Hélio de Almeida Ricardo, Marcelo Corrêa da Silva, Márcio Rodrigues de Souza, Fernando Miranda de Vargas Junior

**Affiliations:** 1 Animal Science Department, Universidade Estadual de Maringá, Maringá, Paraná, Brazil; 2 Animal Science Department, Universidade Federal da Grande Dourados, Dourados, Mato Grosso do Sul, Brazil; 3 Animal Science Department, Escola Luiz de Queiroz, Piracicaba, São Paulo, Brazil; Faculty of Animal Sciences and Food Engineering, University of São Paulo, BRAZIL

## Abstract

This study configures a first report regarding the variability of meat quality of locally adapted Pantaneiro sheep depending on different physiological stages and breeding systems. Pantaneiro sheep are raised in Brazil under a tropical wetland ecosystem denominated Pantanal. Twenty-nine Pantaneiro sheep from different sex and physiological stages were sorted into three groups, simulating three of the most representative ovine meat products commercialized by South American industries: a) non castrated male lambs (n = 11); b) wethers (n = 9); c) cull ewes (n = 9). Animals from each physiological stage were submitted to different breeding systems, resembling farming strategies adopted in several developing countries of South America. The effect of physiological stages on the quality of meat was accessed using 16 variables measured in the *longissimus thoracis et lumborum* (LM) and the *semimembranosus* (SM) muscles. The variables were related to brightness, color, physical aspects, soluble and total collagen as well as chemical traits. The physiological stage was defined as a classification variable in order to proceed ANOVA tests and comparison of means (P<0.05). Multivariate analysis was used to identify patterns of similarity and differentiation between samples of different physiological stages (a, b, c). The results revealed that meat quality varies according to different physiological stages, especially between lambs (a) and cull ewes (c). As a consequence, the physiological stage at slaughter should be taken into consideration to cote the quality of meat from indigenous sheep raised in tropical regions. The results contribute towards sensorial evaluation and the characterization of potential food products derived from indigenous sheep bred under tropical climate in developing countries.

## 1. Introduction

The Midwest of Brazil presents the highest growth rates of sheep farming in the country [1]. This region is an important agricultural frontier, which comprises tropical biomes such as the Brazilian Savannah (Cerrado) and the tropical wetland ecosystem (Pantanal). In these regions sheep farming configures an alternative to increase financial yield and produce food for local markets or exportation.

Several factors limit sheep breeding to play a more significant role on rural development and become a source of financial income in developing countries such as Brazil [2, 3]. The success of sheep farming will rely on tackling different issues that configure bottlenecks which are scattered throughout the production chain and require urgent attendance. Problems are related to great seasonality of tropical pastures, animal health, farming practices or merchandising along the annual calendar [4–7]

Progress has been reported, but market and merchandising of sheep products in many developing countries still contrast with global market trends which requires urgent adjustments [6–13]

There is considerable lack of information regarding breed, geographical provenance, breeding system and traceability of age category (physiological stage), which may denote the quality of meat after slaughter.

Food originated from indigenous breeds of sheep is an option to access and develop alternative markets [14–16]. However, information about locally adapted sheep in South America is rather poor, which includes breeds such as Crioula Lanada [17] and Pantaneiro sheep [18].

It is reasonable to consider that the diversification of products and market niches require efforts towards characterization [2, 19–21]. This is a potential issue to be tackled in the field of science.

The quality of meat has been an important subject [3], including topics related to breed or consumer preferences [3, 6]. Regarding different age categories of sheep (which may be bred in separate groups) the demands are greater for meat originated from lambs. Lambs are considered to present superior quality standards of meat, quite appealing for exportation. Thus, growing and finishing lambs for slaughter has been prioritized in the meat industry.

Still, in countries such as Brazil, three top products derive from sheep farming: meat from lambs (a), wethers (b) and cull ewes (c). Demands for each one of these products vary according to local market trends in different macro regions of South American countries [22]. Physicochemical and sensory aspects of meat may vary between animals of different ages and this is a subject related to consumer preferences and market perspectives [4, 22–25]

Considering some of the aspects of indigenous sheep farming, this study was designed to generate a first report on meat quality of Pantaneiro sheep, encompassing physicochemical and sensory characterization of the most common physiological stages commercialized in South American sheep industries.

## 2. Materials and methods

All protocols of experimental procedures were approved by the Animal Experimentation Ethics Committee (CEUA) of the Federal University of Grande Dourados (UFGD), S1 File, Dourados, State of Mato Grosso do Sul, Brazil. Twenty-nine Pantaneiro sheep from the experimental and on farm conservation flock of UFGD were divided into three physiological stages Table 1. Slaughter ages were defined in order to simulate the most representative products and farming systems in the Brazilian sheep industry. Different farming systems were established according to input priorities related to different physiological stages. Animals were slaughtered and classified as non- castrated lambs (deciduous teeth) (a), wethers (two teeth) (b) and cull ewes (4–8 teeth) (c) Table 1.

**Table 1:** Growth and carcass traits of Pantaneiro sheep raised on different breeding systems, slaughtered based on different physiological stages (a, b, c).

	Physiological stages		
	Lambs (a)	Wethers (b)	Cull ewes (c)
Number of animals	11	9	10
Body weight at birth (kg)	3.6±0.9	3.5±0.7	-
Body weight weaning (kg)	19.0 ±4.0	12.5±1.2	-
Weight gain from birth to slaughter (kg)	0.179±0.3	0.110±0.2	-
Body weight at slaughter (kg)	36.0±4.2	41.8±5.1	48.3±4.6
Body condition at slaughter (1–5)	2.8±0.2	2.9±0.2	3.9±0.4
Age at slaughter (months)	6.2±1.3	12.3±1.2	46.7±5.0
Hot carcass weight (kg)	17.8±2.16	19.8±2.54	23.3±4.05
Cold carcass weight (kg)	17.1±2.15	18.6±2.58	20.8±3.95
Breeding system	*Ad libitum* creep feeding (feedlot) from birth to weaning (60–90 days)	*Ad libitum* creep feeding from birth to weaning (60–90 days) on *Cynodon* spp. (Tifton 85) pasture with concentrate supplementation during the last two months before slaughter	Animals with at least one calving, discarded after negative diagnosis of pregnancy in two consecutive breeding seasons, maintained in pastures of *Brachiaria Brizantha*

(a) non castrated male animals with all deciduous teeth

(b) castrated male animals with exchange of the first pair of incisors

(c) animals with exchange of the medium incisors

The criteria to slaughter lambs (a) and wethers (b) was the body condition, which ranged from 2.5 to 3.0 [26]. Cull ewes (c) were slaughtered based on negative pregnancy diagnosis, regardless of their body condition, which ranged from 4.0 to 4.5 (Table 1).

The uncastrated lambs (a) were weaned with average weight of 19.5±4.10 kg and finished in feedlots with an 80% concentrate and 20% forage (20%) diet (Table 1). The diet was prepared for an average gain of 250 g day^-1^ [27]. Lambs (a) were fed three times a day with commercial concentrate consisted of 16% of crude protein (CP), 70% of total digestible nutrients (TDN), oat hay presenting 7% of CP and 55.64% of TDN. Slaughter of lambs occurred at the age of 6±1 months.

The wethers (b) were kept in tropical pastures based on *Cynodon* spp. (Tifton85) and supplemented (1% of body weight) with the same concentrate used for lambs (water ad libitum). These animals were slaughtered at the age of 12.5±1.18 months. The cull ewes (c) were kept in extensive systems with tropical pastures of *Brachiaria brizantha* cv. Piatã with ad libitum mineral salt. Cull ewes (c) were slaughtered at the age of 68±13 months (Table 1).

All animals (a, b, c) were slaughtered after 16-hours of fasting (solids). Desensitization was carried out by electronarcosis (8 seconds of 220-V discharge) followed by cutting of jugular veins and carotid arteries with subsequent evisceration. Lambs (a), wethers (b) and cull ewes (c) showed initial average weight of 19.64, 19.90 and 45.04 kg, respectively. Final average weight was 35.72, 43.04 and 49.58 kg, respectively Table 1.

### 2.1. Sampling

After slaughter, the carcasses were stored in a cold room at 4°C during 24 hours. Subsequently, the *longissimus thoracis et lumborum* (LM) and *semimembranosus* (SM) muscles were removed for meat quality analysis. All samples were analyzed with one replicate, and the final value of each variable was determined calculating a simple average for each variable.

### 2.2. Instrumental indicators of meat quality

Evaluation of meat was performed based on color, physical characteristics, soluble and total collagen and chemical characteristics Tables 2, 3 and 4.

**Table 2:** Effect of muscle type nested in different physiological stages (M(PhSt)) on the color of meat from indigenous Pantaneiro sheep.

Item^1^	Muscle type	Physiological Stages			CV (%)	P-value	
		lambs (a)	wethers (b)	cull ewes (c)		PhSt	M(PhSt)
L*	*LM*	41.49a ± 1.85	37.13b ± 1.77	35.31b ± 1.36	5.63	<0.0001	0.2470
	*SM*	41.35a ± 2.92	36.85b ± 1.67	33.07c ± 2.57			
a*	*LM*	23.45a ± 1.03	23.16ab ± 0.85	21.46b ± 1.88	7.14	<0.0001	0.5610
	*SM*	24.78a ± 1.38	23.51ab ± 1.95	22.14b ± 1.48			
b*	*LM*	9.44a ± 0.90	8.53ab ± 0.84	7.63b ± 1.23	16.86	<0.0001	0.4290
	*SM*	10.77a ± 1.42	9.13b ± 1.45	8.01b ± 1.28			
Chroma	*LM*	25.29a ± 1.15	24.69ab ± 1.04	22.79b ± 2.04	7.83	<0.0001	0.4980
	*SM*	27.03a ± 1.75	25.23ab ± 2.30	23.56b ± 1.79			
Hue	*LM*	21.92 ± 1.68	20.18 ± 1.37	19.53 ± 2.39	12.22	0.0028	0.5840
	*SM*	23.43a ± 1.97	21.11ab ± 1.82	19.78b ± 1.91			

^1^L*: Brightness; a*: intensity of red; b*: intensity of yellow; Chroma: saturation index; Hue: tonality angle. *LM*: *longissimus thoracis et lumborum*; SM: *semimembranosus;* Low case letters in the lines indicate significant difference (P<0.05) between physiological Stages (PhSt)

**Table 3:** Effect of muscle type nested in different physiological stages (M(PhSt)) on pH, soluble collagen, total collagen and physical characteristics of meat from indigenous Pantaneiro sheep.

Item^1^	Muscle type	Physiological Stages			CV (%)	P-value	
		lambs (a)	wethers (b)	cull ewes (c)		PhSt	M(PhSt)
pH_Final_	*LM*	6.18a ± 0.63	5.60b ± 0.20	6.05a ± 0.46	6.15	<0.0001	0.4963
	*SM*	5.85a ± 0.34	5.47b ± 0.09	6.08a ± 0.49			
CRA, %	*LM*	74.14±3.85	75.17±7.97	74.45±3.76	6.13	0.568	0.067
	*SM*	72.68±3.33	74.04±2.35	70.46±3.55			
SF, kg	*LM*	3.99 ± 0.96	3.65 ± 0.80	2.96B ± 1.07	27.59	0.5064	0.0003
	*SM*	3.45 ± 1.25	4.56 ± 1.13	4.70A ± 1.68			
COL, %	*LM*	2.98aB±0.36	2.29bB ±0.18	2.10bB±0.79	16.04	<0.0001	<0.0001
	*SM*	4.22aA±0.55	3.77abA ±0.45	3.56bA ±0.51			
COLS, %	*LM*	19.81aA± 3.16	18.02ab ± 1.41	16.81b ± 2.03	13.84	0.0154	0.0007
	*SM*	16.17aB±1.74	15.89a ± 2.49	14.67a ±2.63			
PPC, %	*LM*	39.48 ± 5.81	38.64 ± 3.10	38.01 ± 6.13	15.01	0.1211	0.5047
	*SM*	40.16 ± 1.55	36.07 ± 12.51	42.27 ± 4.27			

^1^CRA: water retention capacity; SF: share force; COL: total collagen; COLS: soluble collagen; PPC: cooking losses; *LM*: *longissimus thoracis et lumborum*; SM: *semimembranosus;* Low case letters in the lines indicate significant difference (P<0.05) between physiological Stages (PhSt); Averages presenting upper-case letters in the columns indicate significant difference (P<0.05) regarding the effect of muscle type physiological stages (M(PhSt)).

**Table 4:** Effect of physiological stage (PhSt) and muscle type nested in physiological stages (M(PhSt)) on the chemical composition of meat from indigenous Pantaneiro sheep.

Item^1^	Muscle type	Physiological Stages			CV (%)	P-value	
		lambs (a)	wethers (b)	cull ewes (c)		PhSt	M(PhSt)
Moisture, %	*LM*	75.05aA ± 0.83	72.22b ± 2.97	72.46b ± 1.22	2.05	0.0003	0.0048
	*SM*	72.73 ± 1.37	72.18 ± 0.75	71.69 ± 0.64			
Ashes, %	*LM*	1.09bB ± 0.05	1.12bB ± 0.15	1.27a ± 0.18	10.62	0.0152	<0.0001
	*SM*	1.32A ± 0.13	1.29A ± 0.07	1.36 ± 0.17			
CP, %	*LM*	19.95a ± 2.59	17.69abB ± 3.24	16.44b ± 2.39	16.18	0.0060	0.0268
	*SM*	21.13a ± 3.66	21.80aA ± 2.93	18.14b ± 3.65			
EE, %	*LM*	2.70b ± 1.30	6.08aA ± 3.82	6.19aA ± 1.20	44.94	<0.0001	0.0014
	*SM*	2.13b ± 1.12	3.37abB ± 0.63	3.98aB ± 0.72			

^1^CP: crude protein; EE: ether extract; *LM*: *longissimus thoracis et lumborum*; SM: *semimembranosus;* Low case letters in the lines indicate significant difference (P<0.05) between physiological Stages (PhSt); Averages presenting upper-case letters in the columns indicate significant difference (P<0.05) regarding the effect of muscle type nested in different physiological stages (M(PhSt)).

The color was evaluated in lyophilized samples, submitted to acid digestion, filtration, neutralization, dilution and oxidation for colorimetric reaction. The results were based on the hydroxyproline values from absorbance readings performed in a spectrophotometer with a wavelength of 570 nm. The sample color was evaluated after 30 minutes of exposure to air to allow myoglobin reaction with oxygen [28]. Brightness (L*), red color index (a*) and yellow scale index (b*) were analyzed with a CR-400 colorimeter with illuminant D65, with a viewing angle of 10° and an opening diameter of 8 mm (KONICA MINOLTA Sensing Inc., Japan). The amount was expressed according to the CIELAB color system. The hue angle (h*) was calculated according to the equation h* = tan-1(b*/a*), and the saturation index (c*) was measured using the equation c* = (a*2 + b*2) 0.5 [29].

The physical characteristics of meat were evaluated regarding water retention capacity (%) according to the method described by [30]. The shear force (SF) (kg) of samples was evaluated in accordance to [31] using a texture analyzer (TA-XT2i) with a Warner-Bratzler blade. Weight loss after cooking (%) was estimated according to the difference between the weight of raw and cooked samples which was measured to allow share force evaluation.

The pH and temperature of the warm and the cold carcass (initial pH and time versus final pH after 24 hours) were monitored using a digital thermometer and pH-meter with a Testo 205/206 penetration probe. The determination of the soluble and total collagen content (%) followed the method described by [32] modified by [33]. In this method the collagen content and the fractions were assessed by quantification of the hydroxyproline amino acid and the samples for the soluble collagen content were boiled in a water bath at 80°C during 75 minutes, centrifuged at 4000 rpm during 10 minutes at 20°C. The conversion factor used was 7.14-fold hydroxyproline concentration.

Regarding the chemical composition of meat, moisture was evaluated according to the method 950.46 [34]. Total nitrogen was evaluated according to the Kjeldahl-micro method (928.080; [34]), and crude protein was evaluated based on the total nitrogen content multiplied by the factor 6.25. The ether extract was evaluated according to the method 960.39 and ash evaluation was carried out according to method 920.153 [34].

### 2.3. Statistical analysis

The experimental design was arranged as an exploratory study. Animals were sampled from an on farm conservation flock and sorted according to pre-established physiological stages. The Shapiro-Wilk test was performed to verify the residue data normality, and the Bartlett test was performed to verify homogeneity of variances. Similar to a nested analysis approach, the effect of physiological stages (fixed effect) (lambs (a), wethers (b) and cull ewes(c)) was evaluated on the *longissimusthoracis et lumborum* (LM) and *semimembranosus* (SM) muscles. This was done using a univariate approach (PROC GLM) (p<0.05). Analyses were performed using the software SAS 9.2 (SAS Institute, Cary, NC, USA). A multivariate method was used to standardize data (PROC STANDARD procedure) and to develop clustering analysis (PROC FASTCLUS procedure) assuming three clusters.

## 3. Results

### 3.1. Evaluation of brightness and color of meat

The meat from lambs (a) showed different values compared with cull ewes (c) with only one exception (tonality (hue angle) of the LM muscle Table 2. Lambs showed greater brightness of meat compared with wethers (b) in both muscles (LM and SM) and more intense yellow compared to the wethers (b) in the SM muscle Table 2. There was no color difference (P>0.05) between muscle types (LM *versus* SM) regarding each physiological stage (Table 2). Means between different physiological stages observed in the intensity of red (a*) showed similar variation compared to the intensity of yellow (b*) Table 2.

### 3.2. Evaluation of physical aspects of meat and collagen

Water retention capacity, shear force and losses of weight after cooking presented similar averages (P>0.05) between physiological stages Table 3. Shear force varied from 2.96 to 4.70 and pH varied from 5.5 to 6.2 Table 3. The total content of collagen of the LM muscle differed between lambs (a) and wethers (b) Table 3. The results between soluble collagen of lambs and wethers were similar but there was a difference between lambs and cull ewes regarding the LM muscle Table 3.

The highest concentration of total collagen was found in the meat from cull ewes. Physiological stages presented different values of total collagen Table 3, while lambs presented different soluble collagen compared to wethers and cull ewes. The LM muscle presented greater soluble collagen contents, however not affecting the shear force of either muscle types Table 3.

### 3.3. Evaluation of chemical aspects of meat

All variables related to chemical composition were effected (P<0.05) by the physiological stage. The chemical analysis showed significant differences between means of lambs and cull ewes, except for the ash content of the SM muscle.

The meat from wethers showed the same ether extract content than the cull ewes, regardless of the muscle type Table 4. The LM muscle of lambs presented lower ether extract (P<0.05) compared to wethers and cull ewes. The SM muscle presented lower contents (P<0.05) compared with the cull ewes. This may be relevant not only for the consumption but also for the purpose of choosing meat for cooking different dishes.

Lambs showed greater moisture content in the LM muscle Table 4. Wethers presented lower contents of protein in the LM muscle Table 4. The ether extract content differed in both muscle types of wethers and cull ewes (P<0.05) Table 4. This was not observed in muscle types of lambs (P>0.05).

Considering all 15 variables used to evaluate the quality of meat from different physiological stages, only three variables (water retention capacity, shear force and cooking losses (Table 3) showed no variation regarding any of the muscle types.

### 3.4. Multivariate statistics

Joint analyses of all variables (Table 5) originated a larger cluster consisted of 16 samples (cluster 2) (55.17%) and two smaller clusters built up by seven samples (cluster 1) (24.14%) and six samples (cluster 3) (20.69%), respectively. All samples of cull ewes (c) were classified into the same group (cluster 3), denoting a peculiar identity (fine discrimination) of meat quality Table 5. Cluster 2 consisted of five wethers and eleven 11 lamb samples Table 5. Cluster 1 consisted of four wethers and three lamb samples Table 5.

**Table 5:** Similarity of color, physical and chemical aspects of the *longissimus thoracis et lumborum* and the *semimembranosus* muscle of indigenous Pantaneiro sheep from different physiological stages.

Grouping based on different physiological stages assuming three clusters				
Cluster	Physiological Stage			Total
*f* (%)	lambs* (a)	wethers** (b)	cull ewes*** (c)	
1	0 0.00 0.00 0.00	4 13.79 57.14 44.44	3 10.34 42.86 33.33	7 (24.14)
2	11 37.93 68.75 100.00	5 17.24 31.25 55.56	0 0.00 0.00 0.00	16 (55.17)
3	0 0.00 0.00 0.00	0 0.00 0.00 0.00	6 20.69 100.00 66.67	6 (20.69)
Total	11 37.93	9 31.03	9 31.03	29 (100.00)

*(a) all deciduous teeth

**(b) exchange of the first pair of incisors

***(c) exchange of medium incisors; (Table 1).

Multivariate analysis revealed that meat of some wethers (b) may present similar aspects to the meat of lambs (a). The meat of some cull ewes (c) maybe similar to the meat of wethers (b). However, no cull ewes (c) presented similar meat quality compared to lambs (a) Table 5.

## 4. Discussion

In general, the variables related to brightness (L*), the intensity of yellow (b*) and red (a*) varied according to physiological stages Table 2. The variation of brightness and color between meat from different physiological stages contrasts with some reports comparing different sheep breeds [3]. However, some are in accordance with results reported by [22]. Color analyses confirmed that meat from younger animals show brighter color with stronger color, tonality and saturation [35, 36]. These indicators are related to the perception of consumers regarding freshness and quality of meat [37]. Brightness is expected in muscles of younger animals and is an issue related to marketing and propaganda [38, 39].

There is a trend to believe that many physical aspects of meat vary according to the age of animals at slaughter [5, 40]. Aging domestic sheep before slaughter is usually related to meat quality which may be associated to the distance of muscle filaments (myofibrillar protein), collagen deposition or dimension of collagen fiber, maturation of connective tissues or intramuscular fat. Many of these characteristics are involved with meat texture such as water retention, collagen and maturation of the connective tissue [41–44]

Carcasses from older animals are associated to greater stability and texture of muscle fiber showing less soluble collagen [45]. The same is observed in carcasses from male specimens which may show greater intramuscular connective tissue due to testosterone [46]. Castration may play a role on meat texture but this was not tested in the scope of the present paper.

In general, meat from different physiological stages showed appropriate quality values of collagen and pH, configuring soft meat Table 3. However, the different concentrations of soluble collagen (COLS,%) and total collagen (COL,%) between physiological stages (Table 3) could not be linked to variation of shear force. Attempts to explain variation of shear force were limited to comparisons between muscle types (LM, SM) (Table 3, (c)).

The chemical characteristics of meat also differed between physiological stages Table 4. The fat contents we observed were fairly within ideal contents reported by [47] (around 3 to 7%) Table 4. This is most likely due to the decrease of moisture and fat content associated to puberty [48, 49]. Moreover, the end of puberty is associated to a gradual increase of adipose tissue and loss of muscle tissue (protein) (Table 4). Thus, meat from animals derived from more advanced physiological stages tend to present greater ash content, as observed in cull ewes Table 4. The subcutaneous fat and muscle accumulation are also important aspects regarding merchandising [50, 51]. The ether extract content may vary according to the muscle type. Higher contents of fat (adipose tissue) are expected in the loin and the breast compared to muscles from the shoulder and shank (locomotor system) [52].

The variation of meat quality regarding muscle types was considered to be random. However, diversifying aspects related to flavor and nutritional aspects of indigenous sheep meat was observed between different physiological stages.

Fine discrimination observed with multivariate analysis Table 5 (a versus c) denoted that the quality of indigenous Pantaneiro meat may vary according to physiological stages, consistent with expectations regarding animal physiology. This should be a subject for merchandising and market perspectives.

## 5. Conclusion

Evaluation of meat from indigenous Pantaneiro sheep of different sex and physiological stages revealed suitable standards of meat quality produced in a rough and typically unique tropical ecosystem. Notable differences were observed between the quality of meat originated from lambs and cull ewes. This fine discrimination opens the door to valuable validation of how meat quality relates to sensorial aspects and consumer preferences regarding the preparation of dishes and consumption.

Characterization of food products derived from locally adapted livestock in developing countries is an effort towards the development of new products with added value which may enhance merchandising, marketing strategies and consumption.

## Supporting information

S1 File Animal Experimentation Ethics Committee (CEUA). Protocols of experimental procedures were approved by commuittes
(PDF)
