# Supplementary material for: Different physiological stages and breeding systems related to the variability of meat quality of indigenous Pantaneiro sheep
Source: PLoS One. 2018 Feb 12;13(2):e0191668. doi: 10.1371/journal.pone.0191668 (PMC5809010; doi:10.1371/journal.pone.0191668)
Supplement: S1 File — Protocols of experimental procedures were approved by commuittes (PDF) [file pone.0191668.s001.pdf]

MINISTÉRIO DA EDUCAÇÃO
FUNDAÇÃO UNIVERSIDADE FEDERAL DA GRANDE DOURADOS
PRÓ-REITORIA DE ENSINO DE PÓS-GRADUAÇÃO E PESQUISA

COMISSÃO DE ÉTICA NO USO DE ANIMAIS - CEUA

Dourados-MS, 20 de março de 2013

Senhor Pesquisador:

Fernando Miranda de Vargas Junior

O Projeto de sua responsabilidade – Protocolo nº. **007/2013 – CEUA/UFGD** - intitulado **"Qualidade da carne de diferentes categorias de ovinos `Pantaneiros´"** foi integralmente **APROVADO** e poderá ser conduzido.

Ressaltamos que é de responsabilidade do (a) pesquisador (a) envio de notificação à CEUA sobre o término do projeto.

Felipe de Almeida Borges
Secretário CEUA/UFGD

Felipe de Almeida Borges
Assistente em Administração
SIAPE - 1669836

[illegible]
